# FDA-MIMO Radar Rapid Target Localization via Reconstructed Reduce Dimension Rooting

**DOI:** 10.3390/s25020513

**Published:** 2025-01-17

**Authors:** Cheng Wang, Zhi Zheng, Wen-Qin Wang

**Affiliations:** 1 Yangtze Delta Region Institute (Huzhou), University of Electronic Science and Technology of China, Huzhou 313002, China; chengw@csj.uestc.edu.cn; 2 School of Electronic Science and Engineering, University of Electronic Science and Technology of China, Chengdu 611731, China; wqwang@uestc.edu.cn; 3 Yangtze Delta Region Institute (Quzhou), University of Electronic Science and Technology of China, Quzhou 324000, China

**Keywords:** FDA-MIMO radar, rapid target localization, spectrum reconstruction, RDRR-MUSIC

## Abstract

Frequency diversity array–multiple-input multiple-output (FDA-MIMO) radar realizes an angle- and range-dependent system model by adopting a slight frequency offset between adjacent transmitter sensors, thereby enabling potential target localization. This paper presents FDA-MIMO radar-based rapid target localization via the reduction dimension root reconstructed multiple signal classification (RDRR-MUSIC) algorithm. Firstly, we reconstruct the two-dimensional (2D)-MUSIC spatial spectrum function using the reconstructed steering vector, which involves no coupling of direction of arrival (DOA) and range. Subsequently, the 2D spectrum peaks search (SPS) is converted into one-dimensional (1D) SPS to reduce the computational complexity using a reduction dimension transformation. Finally, we conduct polynomial root finding to further eliminate computational costs, in which DOA and range can be rapidly estimated without performance degradation. The simulation results validate the effectiveness and superiority of the proposed RDRR-MUSIC algorithm over the conventional 2D-MUSIC algorithm and reduced-dimension (RD)-MUSIC algorithm.

## 1. Introduction

The multiple-input multiple-output (MIMO) radar fully utilizes space diversity to improve direction estimation accuracy, communication capacity, and interference suppression capability, which has been widely adopted in sonar, satellite navigation, and mobile communications [1,2]. However, the MIMO radar system model is independent of the target range, which limits the applications of the MIMO radar. Fortunately, the frequency diversity array (FDA) employs a slight frequency offset between adjacent transmitter sensors to realize the angle- and range-dependent beam pattern [3,4,5], which enjoys the potential of joint angle and range estimation. In order to fully utilize the advantages of the MIMO radar and FDA, a new radar system was investigated in [6,7,8], which merged the FDA and MIMO radar into a hybrid named the FDA-MIMO radar. The FDA-MIMO radar significantly improves the degrees of freedom (DOFs) of target localization, which has drawn much research attention. However, the ambiguous estimates caused by the coupling of the angle and range limit the applications of the FDA-MIMO radar. Researchers have proposed several schemes to solve this problem.

A double pulses scheme has been proposed to solve the coupling of the direction of arrival (DOA) and range [9], in which a pulse with zero frequency offset is utilized to estimate the DOA, and another pulse with non-zero frequency offset is exploited to estimate the range without coupling. However, the double pulses scheme adopts the maximum likelihood (ML) algorithm to estimate the DOA and range, which involves a high computational burden. In [10], a transmitter subaperture-based scheme was studied, in which the transmitter is divided into several subtransmitters to erase the coupling, and the DOA and range are successively estimated. However, the DOA and range are estimated by searching the 2D spatial domain, which restricts the practical uses of this transmitter subaperture-based scheme. Estimation of signal parameters via rotational invariance techniques (ESPRIT)-based algorithms [11,12,13] utilize the rotational invariance property of the structure to rapidly estimate the DOA and range without coupling, but the estimation accuracy needs to be further improved. The conventional 2D-MUSIC algorithm [14] can accurately estimate DOA and range using the 2D spectrum peaks search (SPS), which avoids an extra decoupling operation, but the computational complexity increases exponentially with an increasing number of search grids. The RD-MUSIC algorithm [15] exploits the reduction dimension transformation (RDT) to decouple DOA and range, and thereby reduces the computational complexity, but the DOA information is not fully utilized, resulting in DOA estimation performance degradation. Meanwhile, the error conduction caused by the extra decoupling operation also deteriorates the range estimation performance. In general, the algorithms mentioned above all require a trade-off between estimation reliability and estimation effectiveness.

In this paper, we propose the reduction dimension root reconstructed MUSIC (RDRR-MUSIC) algorithm to rapidly estimate DOA and range. We first reconstruct the 2D-MUSIC spatial spectrum function (SSF), utilizing the reconstructed steering vector without coupling the DOA and range. Then, we convert 2D SPS into 1D SPS using RDT; therefore, the computational complexity is significantly reduced. Finally, 1D SPS is transformed into polynomial root finding (PRF) to further reduce the computational complexity, and the DOA and range are rapidly estimated, but with no performance degradation. The advantages of the proposed RDRR-MUSIC algorithm are demonstrated using numerical simulation.

The contributions of this paper are as follows:

(1) We decouple DOA and range by reconstructing the steering vector, and all DOA information is fully used in PRF, which prevents DOA estimation performance degradation.
(2) We construct a transformation mechanism between DOA and range, and eliminate the error conduction caused by the extra decoupling operation, which prevents range estimation performance degradation.
(3) We achieve the automatic pairing of DOA estimates and range estimates.
(4) We reduce the large computational costs and realize rapid target localization using RDT and PRF.

The remainder of this paper can be summarized as follows: Section 2 shows the system model. The 2D-MUSIC SSF reconstruction, RDT, and PRF are presented in Section 3. Section 4 comprises the numerical simulations. The conclusion can be found in Section 5.

## 2. System Model

**Figure 1:**
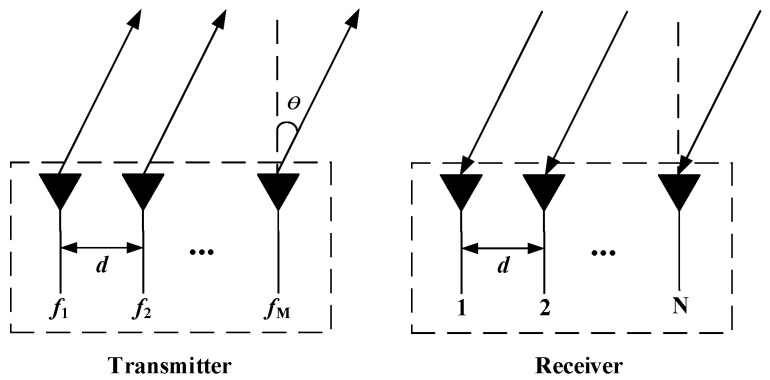
Collocated FDA-MIMO radar.

As shown in Figure 1, the transmitter and receiver of collocated FDA-MIMO radar contain *M* and *N* sensors, respectively, and they are all uniform linear arrays with d=λ/2 interspace, where λ is the wavelength. The radiated frequency of the *m*th sensor is defined as follows:

(1) $$f_{m}=f_{0}+\left(m-1\right)\Delta f,m=1,2,\ldots ,M$$

where $f_{0}$ is the carrier frequency, Δf is the frequency offset between adjacent sensors, and $\Delta f\ll f_{0}$. We assume that *K* point targets need to be located, and $\left(\theta_{k},r_{k}\right)$ are the DOA and range of the *k*th target, k=1,2,…,K. Then, the output signal after matched filtering can be given as follows: [6]

(2) x(t)=As(t)+n(t)

where $s\left(t\right)={\left[s_{1}\left(t\right),s_{2}\left(t\right),\ldots ,s_{K}\left(t\right)\right]}^{T}$ is the signal vector, $s_{k}\left(t\right)=\sigma_{k}e^{j2\pi f_{d_{k}}\left(t-\tau_{0}\right)}e^{-4\pi f_{0}r_{k}/c}$, $\sigma_{k}$ is the radar cross section, $f_{d_{k}}$ is the Doppler frequency, $\tau_{0}=2r/c$ is the time delay between the first sensor and target, *c* is light speed, and $n\left(t\right)∼\mathrm{CN}\left(0,\delta^{2}I\right)$ is the noise vector. Moreover, A is the steering matrix which is given as follows:

(3) $$\begin{array}{cc} A & =\left[a_{r}\left(\theta_{1}\right)⊗a_{t}\left(\theta_{1},r_{1}\right),\ldots ,a_{r}\left(\theta_{K}\right)⊗a_{t}\left(\theta_{K},r_{K}\right)\right]\\ \; & =\left[a\left(\theta_{1},r_{1}\right),\ldots ,a\left(\theta_{K},r_{K}\right)\right] \end{array}$$

where $a(\theta_{k},r_{k})$ is the steering vector, ⊗ is the Kronecker product, and $a_{r}\left(\theta_{k}\right)$ is the receive steering vector, which is defined as follows:

(4) $$a_{r}\left(\theta_{k}\right)={\left[1,e^{j2\pi d\sin\left(\theta_{k}\right)/\lambda },\ldots ,e^{j2\pi \left(N-1\right)d\sin\left(\theta_{k}\right)/\lambda }\right]}^{T}$$

$a_{t}\left(\theta_{k},r_{k}\right)$ is the transmit steering vector, which is defined as follows:

(5) $$a_{t}\left(\theta_{k},r_{k}\right)=a_{t}\left(\theta_{k}\right)⊕a_{t}\left(r_{k}\right)$$

⊕ is the Hadamard product and $a_{t}\left(\theta_{k}\right)$ is the DOA-dependent transmit steering vector, defined as follows:

(6) $$a_{t}\left(\theta_{k}\right)={\left[1,e^{j2\pi d\sin\left(\theta_{k}\right)/\lambda },\ldots ,e^{j2\pi \left(M-1\right)d\sin\left(\theta_{k}\right)/\lambda }\right]}^{T}$$

$a_{t}\left(r_{k}\right)$ is the range-dependent transmit steering vector, defined as follows:

(7) $$a_{t}\left(r_{k}\right)={\left[1,e^{-j4\pi \Delta fr_{k}/c},\ldots ,e^{-j4\pi \left(M-1\right)\Delta fr_{k}/c}\right]}^{T}$$

When we consider finite samples, the covariance matrix (CM) of the output signal can be presented as follows:

(8) $$\hat{R}=\frac{1}{L}\sum_{l=1}^{L}x\left(l\right)x^{H}\left(l\right)$$

where *L* is the snapshot.

## 3. Proposed Method

We conduct eigenvalue decomposition (CED) for $\hat{R}$, resulting in the following:

(9) $$\hat{R}=E_{s}D_{s}E_{s}^{H}+E_{n}D_{n}E_{n}^{H}$$

where $E_{s}\in C^{NM\times K}$ is the signal subspace containing the eigenvectors corresponding to the largest *K* eigenvalues and $E_{n}\in C^{NM\times \left(NM-K\right)}$ is the noise subspace that consists of eigenvectors corresponding to the other eigenvalues [16]. We construct 2D-MUSIC SSF as in [15], as follows:

(10) $$f_{2D-MUSIC}\left(\theta ,r\right)=\frac{1}{a^{H}\left(\theta ,r\right)E_{n}E_{n}^{H}a\left(\theta ,r\right)}$$

We can find *K* spectrum peaks using 2D SPS, and the entries of the peaks denote the locations of the targets. However, the conventional 2D-MUSIC algorithm cannot be utilized directly in practice due to the heavy computational burden caused by 2D SPS. Therefore, we propose the RDRR-MUSIC algorithm to overcome this drawback.

We reconstruct the steering vector with the decoupling of DOA and range, as follows:

(11) $$\begin{array}{cc} a(\theta ,r) & =a_{r}\left(\theta \right)⊗\left[\mathrm{diag}\left(a_{t}\left(\theta \right)\right)a_{t}\left(r\right)\right]\\ \; & =\left[a_{r}\left(\theta \right)⊗\mathrm{diag}\left(a_{t}\left(\theta \right)\right)\right]a_{t}\left(r\right)\\ \; & =D\left(\theta \right)a_{t}\left(r\right) \end{array}$$

where diag(.) represents a diagonal matrix where the diagonal entries are composed of the entries of a vector. Note that $a_{r}\left(\theta \right)$ and $a_{t}\left(\theta \right)$ are combined into D(θ), which is independent of the range. The 2D-MUSIC SSF can be reconstructed as follows:

(12) $$f_{RC-MUSIC}\left(\theta ,r\right)=\frac{1}{a_{t}^{H}\left(r\right)D^{H}\left(\theta \right)E_{n}E_{n}^{H}D\left(\theta \right)a_{t}\left(r\right)}$$

Then, we conduct RDT, to alleviate the computational complexity.

We construct the optimization function as in [15], as follows:

(13) $$\min_{\theta ,r}a_{t}^{H}\left(r\right)Q\left(\theta \right)a_{t}\left(r\right)\;\;s.t.\;e_{1}^{H}a_{t}\left(r\right)=1$$

where $Q\left(\theta \right)=D^{H}\left(\theta \right)E_{n}E_{n}^{H}D\left(\theta \right)$, $e_{1}={\left[1,0,\ldots ,0\right]}^{T}\in R^{M\times 1}$. Then, the loss function is as in [15], as follows:

(14) $$L\left(\theta ,r\right)=a_{t}^{H}\left(r\right)Q\left(\theta \right)a_{t}\left(r\right)-\eta \left(e_{1}^{H}a_{t}\left(r\right)-1\right)$$

where η is the regularization coefficient. Then, we can obtain the following:

(15) $$\frac{\partial L(\theta ,r)}{\partial a_{t}\left(r\right)}=2Q\left(\theta \right)a_{t}\left(r\right)-\eta e_{1}=0$$

We consider $e_{1}^{H}a_{t}\left(r\right)=1$; therefore, we can obtain the following:

(16) $$a_{t}\left(r\right)=\frac{Q^{-1}\left(\theta \right)e_{1}}{e_{1}^{H}Q^{-1}\left(\theta \right)e_{1}}$$

Note that the transformation mechanism between DOA and range is constructed, and the extra decoupling operation is undesired because $a_{t}\left(r\right)$ is independent from DOA. The optimization function can be further reconstructed as follows:

(17) $$\hat{\theta }=\min_{\theta }\frac{1}{e_{1}^{H}Q^{-1}\left(\theta \right)e_{1}}=\min_{\theta }\frac{\det(Q(\theta ))}{\mathrm{compan}{\left(Q\left(\theta \right)\right)}_{1,1}}$$

where det(.) is the determinant and compan(.) is the companion matrix. We can estimate the DOA by searching the zero value of det(Q(θ)), and the 2D SPS is converted into 1D SPS using RDT. In order to further reduce the computational complexity, we construct the following symbol vector:

(18) $$\begin{array}{cc} Q(z) & ={\left[z^{N-1}a_{r}\left(z^{-1}\right)⊗\mathrm{diag}\left(z^{M-1}a_{t}\left(z^{-1}\right)\right)\right]}^{T}E_{n}\\ \; & \times E_{n}^{H}\left[a_{r}\left(z\right)⊗\mathrm{diag}\left(a_{t}\left(z\right)\right)\right] \end{array}$$

where $z=e^{j2\pi d\sin(\theta )/\lambda }$ is the symbol variable, $z^{N-1}$ and $z^{M-1}$ are utilized to avoid negative power series of *z*, $a_{r}\left(z\right)={\left[1,z,\ldots ,z^{N-1}\right]}^{T}$, and $a_{t}\left(z\right)={\left[1,z,\ldots ,z^{M-1}\right]}^{T}$. We can find *K* roots ${\hat{z}}_{1},{\hat{z}}_{2},\ldots ,{\hat{z}}_{K}$ by conducting PRF to det(Q(z))=0, and the DOA estimates ${\hat{\theta }}_{k}$ can be given as follows:

(19) $${\hat{\theta }}_{k}=\arcsin\left[\mathrm{angle}\left({\hat{z}}_{k}\right)\lambda /2\pi d\right],\;k=1,2,\ldots ,K$$

where angle(.) is the phase angle of a complex number.

Considering Equation (16), we obtain the following:

(20) $${\hat{a}}_{t}\left(r_{k}\right)=\frac{Q^{-1}\left({\hat{\theta }}_{k}\right)e_{1}}{e_{1}^{H}Q^{-1}\left({\hat{\theta }}_{k}\right)e_{1}}$$

We construct the least square fitting (LSF) of the range, as follows:

(21) $${\hat{g}}_{k}=\min_{g_{k}}{∥Gg_{k}-\mathrm{angle}\left({\hat{a}}_{t}\left(r_{k}\right)\right)∥}^{2},k=1,2,\ldots ,K$$

where $G=[1_{M},\mu ]$, $\mu ={\left[0,-4\pi \Delta f/c,\ldots ,-4\pi \left(M-1\right)\Delta f/c\right]}^{T}$, $1_{M}={\left[1,1,\ldots ,1\right]}^{T}\in R^{M\times 1}$, $g_{k}={\left[\epsilon_{k},r_{k}\right]}^{T}$, and $\epsilon_{k}$ is residual. The range estimates ${\hat{r}}_{k}$ can be given as follows:

(22) $${\hat{g}}_{k}={\left(G^{H}G\right)}^{-1}G^{H}\mathrm{angle}\left({\hat{a}}_{t}\left(r_{k}\right)\right),k=1,2,\ldots ,K$$

where ${\hat{r}}_{k}$ is the second element of ${\hat{g}}_{k}$.

**Remark 1.**  *The transformation mechanism between the DOA and the range is constructed as Equation (16), so the DOA estimates and range estimates are automatically paired.*

**Remark 2.**  *The computational complexity of the proposed RDRR-MUSIC algorithm includes the following parts: the CM construction in Equation (8) requires $O({\left(MN\right)}^{2}L)$, the CED in Equation (9) needs $O({\left(MN\right)}^{3})$, the symbol vector construction and PRF in Equation (18) requires $O(M^{2}\left(MN-K\right)+{\left(2M\left(N-1\right)\right)}^{3})$, and LSF in Equation (21) needs O((3M+1)K), which avoids the heavy computational cost of SPS.*

**Remark 3.**  *The proposed RDRR-MUSIC algorithm can also be utilized in the multipath interference scenario, but the rank of the covariance matrix in Equation (8) needs to be recovered via spatial smoothing [17].*

The specific steps of the proposed RDRR-MUSIC algorithm are concluded as follow Algorithm 1:

**Algorithm 1** RDRR-MUSIC**Input:** The output signal x(t)
1:Calculate R and En using Equations (8) and (9);
2:Reconstruct 2D-MUSIC SSF utilizing reconstructed steering vector through Equation (12);
3:Perform RDT and PRF to obtain DOA estimates using Equation (19);
4:Perform LSF to obtain range estimates using Equation (21);
**Output:**
$({\hat{\theta }}_{k},{\hat{r}}_{k}),k=1,2,\ldots ,K$


## 4. Complexity Analysis

**Figure 2:**
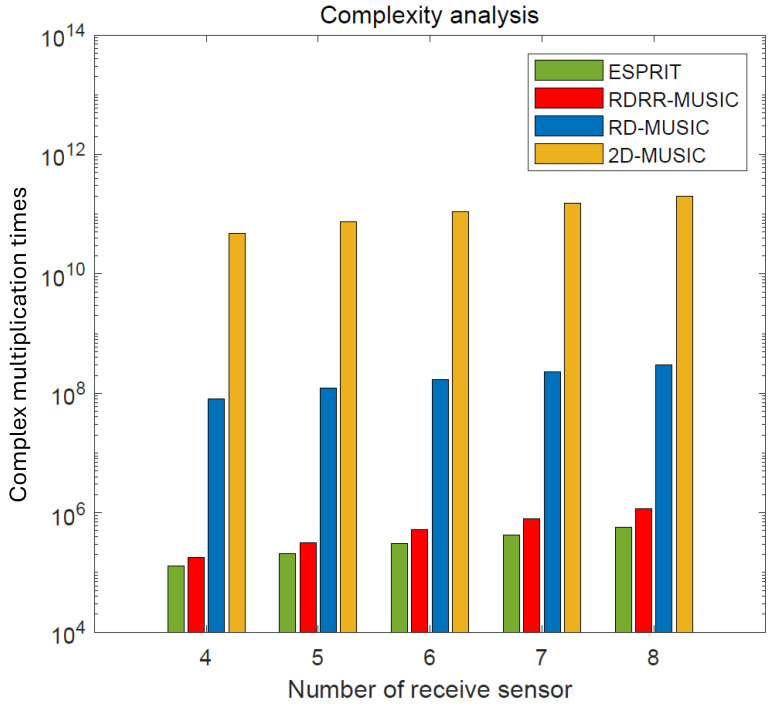
Complexity comparison versus the number of receive sensors.

**Figure 3:**
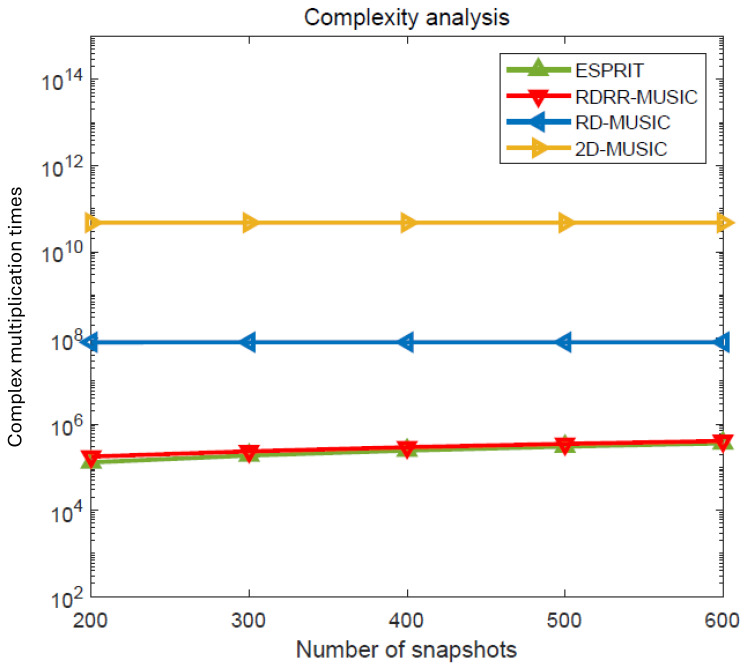
Complexity comparison versus the number of snapshots.

The computational complexity of the proposed RDRR-MUSIC algorithm, the ESPRIT algorithm [11], the conventional 2D-MUSIC algorithm [14], and the RD-MUSIC algorithm [15] are described in Table 1, where Δθ and Δr are the DOA and range search scopes, and $\theta_{d}$ and $r_{d}$ are the DOA and range search step widths, respectively. Meanwhile, the complexity comparison versus the number of receive sensors is presented in Figure 2, where M=6, L=200, $\Delta \theta =180^{{}^{\circ}}$, Δr=500 m,$\theta_{d}=0.01{}^{\circ}$, and $r_{d}=0.01$ m. The complexity comparison versus the number of snapshots is shown in Figure 3, where M=6, N=4, $\Delta \theta =180^{{}^{\circ}}$, Δr=500 m, $\theta_{d}=0.01{}^{\circ}$, and $r_{d}=0.01$ m. It is obvious that the computational complexity of the proposed RDRR-MUSIC is much lower than that of the conventional 2D-MUSIC algorithm and RD-MUSIC algorithm, and is almost the same as for the ESPRIT algorithm. The reason for this is that 2D SPS and 1D SPS are successfully eliminated by RDT and PRF.

In order to further demonstrate the effectiveness of the proposed RDRR-MUSIC algorithm, a comparison of the actual computation time is shown in Table 2, where M=3, N=3, L=400, $\Delta \theta =180^{{}^{\circ}}$, Δr=500 m, $\theta_{d}=0.01{}^{\circ}$, and $r_{d}=0.01$ m. Different algorithms are computed by the MATLAB R2021b with Intel Core i5-12400 @2.5 GHz and 16GB RAM. We found that the computation time of the proposed RDRR-MUSIC algorithm is shorter than that of the conventional 2D-MUSIC algorithm [14] and the RD-MUSIC algorithm [15].

## 5. Numerical Simulations

We perform numerical simulations to validate the superiority and effectiveness of the proposed RDRR-MUSIC algorithm. Different algorithms are compared with the proposed RDRR-MUSIC, including the ESPRIT algorithm [11], the conventional 2D-MUSIC algorithm [14], the RD-MUSIC algorithm [15], and the Cramér–Rao bound (CRB). The root mean square error (RMSE) is utilized to evaluate all the algorithms mentioned above and is defined as follows:

(23) $$RMSE_{\beta }=\sqrt{\frac{1}{KP}\sum_{k=1}^{K}\sum_{p=1}^{P}{\left({\hat{\beta }}_{k,p}-\beta_{k}\right)}^{2}}$$

where ${\hat{\beta }}_{k,p}$ is the DOA or range estimate of the *k*th target, which is conducted by the *p*th trail, $\beta_{k}$ is the real DOA or range of the *k*th target, and *P* denotes the number of Monte Carlo trials. Table 3 shows the simulation parameter configuration, where $\Delta f\ll f_{0}$. Note that the maximum unambiguous estimation range is $r_{max}=c/2\Delta f$ [6].

**Figure 4:**
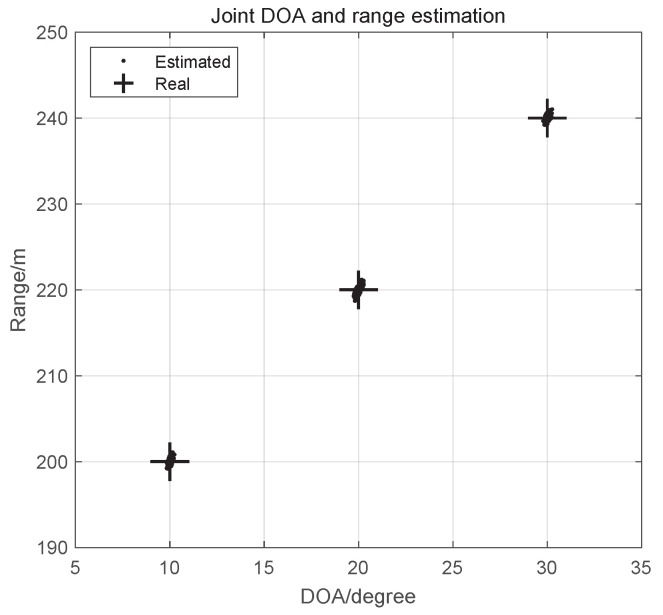
Scatter figure.

**Example 1.**  *The estimates of the proposed RDRR-MUSIC algorithm are shown in Figure 4, where the signal-to-noise ratio (SNR) is SNR=5 dB, L=400, and P=200. We found that the estimates of three targets are close to the corresponding real values, and the DOA estimates and range estimates are automatically paired, which verifies the reliability of the proposed RDRR-MUSIC algorithm.*

**Figure 5:**
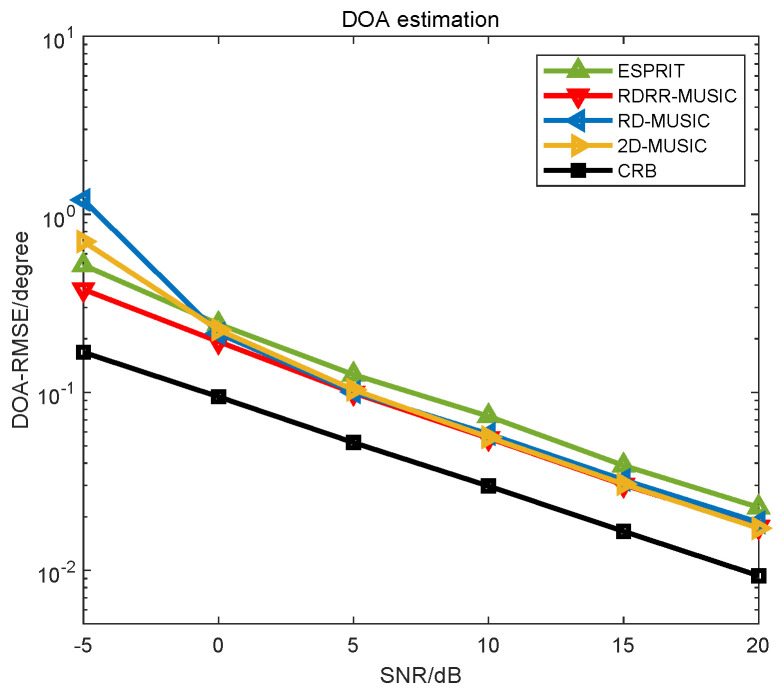
DOA estimation performance comparison of different algorithms versus SNR.

**Figure 6:**
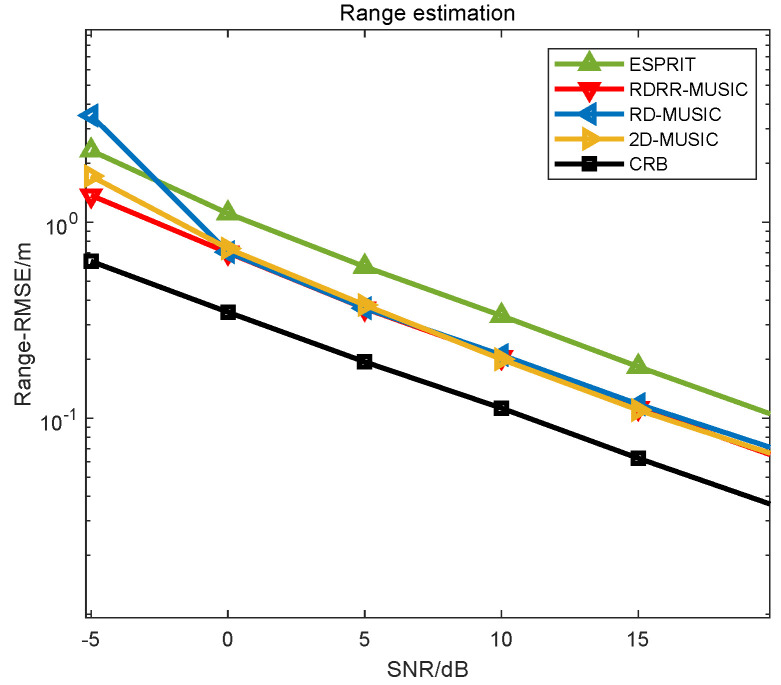
Range estimation performance comparison of different algorithms versus SNR.

**Example 2.**  *The performance comparison of different algorithms versus SNR are presented in Figure 5 and Figure 6, where L=400, P=600$,\theta_{d}=0.01{}^{\circ}$, and $r_{d}=0.01$ m. In Figure 5 and Figure 6, we can observe that the RMSE curves of the proposed RDRR-MUSIC algorithm, the conventional 2D-MUSIC algorithm, and the RD-MUSIC algorithm almost overlap in SNR>0dB cases, which means that they enjoy identical estimation accuracy. In SNR≤0 dB cases, the proposed RDRR-MUSIC algorithm enjoys a better estimation performance than the RD-MUSIC algorithm, because the proposed RDRR-MUSIC algorithm fully exploits all DOA information when conducting PRF, and the error conduction caused by the extra decoupling operation is eliminated. Moreover, the proposed RDRR-MUSIC algorithm outperforms the conventional 2D-MUSIC algorithm in SNR≤0 dB cases. The reason for this is that, because the estimates of the conventional 2D-MUSIC algorithm are generated by 2D SPS, the grid mismatch problem cannot be solved, even in a dense search grid. Meanwhile, the DOA searching error and range searching error will interfere with each other, deteriorating the 2D searching accuracy. However, the estimates of the proposed RDRR-MUSIC algorithm are calculated using PRF, which avoids the grid mismatch problem.*

**Figure 7:**
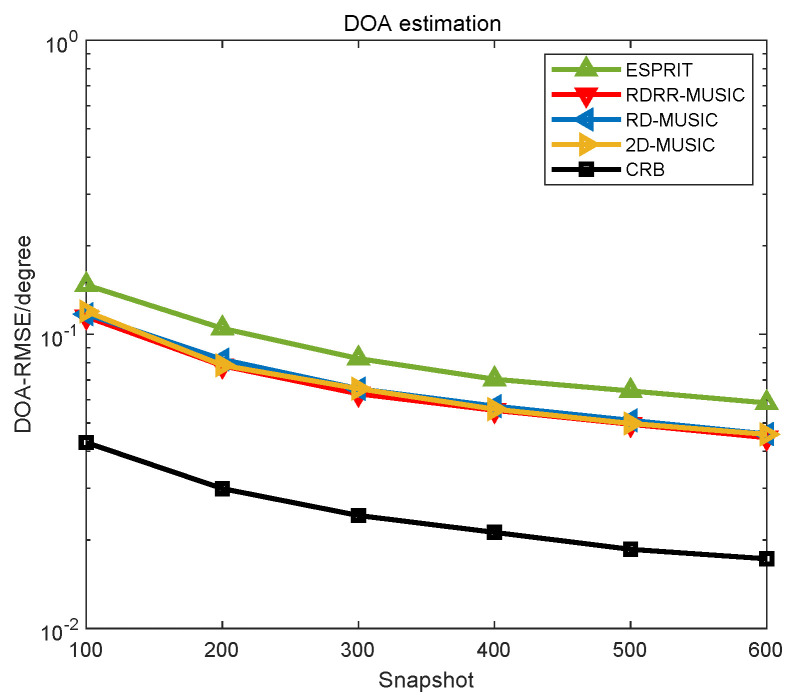
DOA estimation performance comparison of different algorithms versus the number of snapshots.

**Figure 8:**
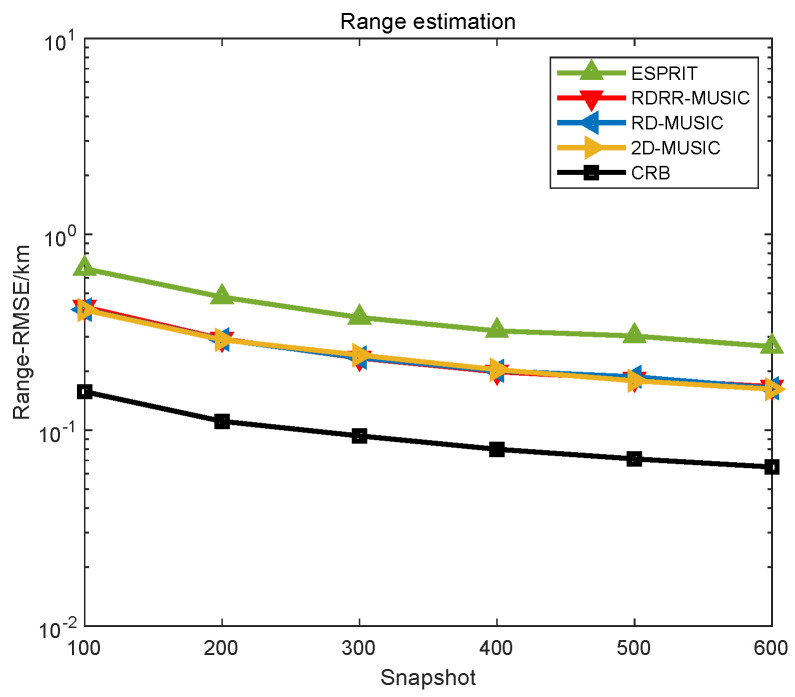
Range estimation performance comparison of different algorithms versus the number of snapshots.

The performance comparison of different algorithms versus the number of snapshots are also plotted in Figure 7 and Figure 8, where SNR=10 dB, P=600, $\theta_{d}=0.01{}^{\circ}$, and $r_{d}=0.01$ m. In Figure 7 and Figure 8, the proposed RDRR-MUSIC algorithm, the conventional 2D-MUSIC algorithm, and the RD-MUSIC algorithm have identical estimation performances, and they all outperform the ESPRIT algorithm. However, the computational complexity of the proposed RDRR-MUSIC algorithm is much lower than that of the conventional 2D-MUSIC algorithm and the RD-MUSIC algorithm, which means that the DOA and range can be rapidly estimated without performance degradation. This simulation demonstrates the superiority of the proposed RDRR-MUSIC algorithm.

## 6. Conclusions

The RDRR-MUSIC algorithm, used to rapidly locate targets using the FDA-MIMO radar, was proposed in this paper. We decoupled the DOA and range by reconstructing the steering vector, and achieved the automatic pairing of DOA estimates and range estimates. Meanwhile, we realized rapid target localization, but without performance degradation. Numerical simulations were used to demonstrate the effectiveness and superiority of the proposed RDRR-MUSIC algorithm. However, the accuracy of the DOA and range estimates were limited by the system’s framework, and the array aperture and signal bandwidth need to be expanded. In future research, we will propose sparse frameworks and the corresponding algorithms to further improve the DOA and range estimation performance.

## Figures and Tables

**Table 1:** Computational complexity comparison of different algorithms.

RDRR-MUSIC	$O({\left(MN\right)}^{2}L+{\left(MN\right)}^{3}+M^{2}\left(MN-K\right)+{\left(2M\left(N-1\right)\right)}^{3}+\left(3M+1\right)K)$
ESPRIT	$O({\left(MN\right)}^{2}L+{\left(MN\right)}^{3}+2K^{2}M\left(N-1\right)+2K^{2}\left(M-1\right)N+6K^{3})$
2D-MUSIC	$O({\left(MN\right)}^{2}L+{\left(MN\right)}^{3}+\left(MN+1\right)\left(MN-K\right)\Delta \theta \Delta r/\theta_{d}r_{d})$
RD-MUSIC	$O({\left(MN\right)}^{2}L+{\left(MN\right)}^{3}+\left(M^{2}\left(N+1\right)\left(MN-K\right)+M^{3}\right)\Delta \theta /\theta_{d})$

**Table 2:** Computation time comparison of different algorithms.

Algorithm	Complexity Multiplications	Computation Time, s
ESPRIT	$3.3507\times 10^{4}$	$0.552\times 10^{-3}$
RDRR-MUSIC	$3.4941\times 10^{4}$	$0.4704\times 10^{-1}$
RD-MUSIC	$4.8931\times 10^{6}$	$0.8895\times 10^{-1}$
2D-MUSIC	$5.4\times 10^{9}$	6.1463

**Table 3:** Parameter configuration.

Parameter	*M*	*N*	$f_{0}$	Δf	*K*	$\left(\theta_{k},r_{k}\right)$
Value	6	8	10 GHz	300 KHz	3	$\left(10^{{}^{\circ}},200\right)$ m $\left(20^{{}^{\circ}},220\right)$ m $\left(30^{{}^{\circ}},240\right)$ m

## Data Availability

The data used to support the findings of this study are available from the corresponding author upon request.
